# Methylene Blue as a Cerebral Metabolic and Hemodynamic Enhancer

**DOI:** 10.1371/journal.pone.0046585

**Published:** 2012-10-09

**Authors:** Ai-Ling Lin, Ethan Poteet, Fang Du, Roy C. Gourav, Ran Liu, Yi Wen, Andrew Bresnen, Shiliang Huang, Peter T. Fox, Shao-Hua Yang, Timothy Q. Duong

**Affiliations:** 1 Research Imaging Institute, University of Texas Health Science Center at San Antonio, San Antonio, Texas, United States of America; 2 Department of Cellular and Structural Biology, University of Texas Health Science Center at San Antonio, San Antonio, Texas, United States of America; 3 Department of Pharmacology and Neuroscience, Institute for Alzheimer’s Disease and Aging Research, University of North Texas Health Science Center at Fort Worth, Fort Worth, Texas, United States of America; 4 Department of Ophthalmology, University of Texas Health Science Center at San Antonio, San Antonio, Texas, United States of America; Banner Alzheimer’s Institute, United States of America

## Abstract

By restoring mitochondrial function, methylene blue (MB) is an effective neuroprotectant in many neurological disorders (e.g., Parkinson’s and Alzheimer’s diseases). MB has also been proposed as a brain metabolic enhancer because of its action on mitochondrial cytochrome *c* oxidase. We used *in vitro* and *in vivo* approaches to determine how MB affects brain metabolism and hemodynamics. For *in vitro*, we evaluated the effect of MB on brain mitochondrial function, oxygen consumption, and glucose uptake. For *in vivo*, we applied neuroimaging and intravenous measurements to determine MB’s effect on glucose uptake, cerebral blood flow (CBF), and cerebral metabolic rate of oxygen (CMRO_2_) under normoxic and hypoxic conditions in rats. MB significantly increases mitochondrial complex I–III activity in isolated mitochondria and enhances oxygen consumption and glucose uptake in HT-22 cells. Using positron emission tomography and magnetic resonance imaging (MRI), we observed significant increases in brain glucose uptake, CBF, and CMRO_2_ under both normoxic and hypoxic conditions. Further, MRI revealed that MB dramatically increased CBF in the hippocampus and in the cingulate, motor, and frontoparietal cortices, areas of the brain affected by Alzheimer’s and Parkinson’s diseases. Our results suggest that MB can enhance brain metabolism and hemodynamics, and multimetric neuroimaging systems offer a noninvasive, nondestructive way to evaluate treatment efficacy.

## Introduction

As powerhouses in mammalian cells, mitochondria are responsible for the predominant mode of energy generation (ATP production) via oxidative phosphorylation of glucose. However, mitochondria are also the major sites for production of reactive oxygen species (ROS), which can cause deleterious effects on cell structure and function if ROS generation significantly exceeds clearance [1]. The imbalance between mitochondrial energy generation and ROS production leads to degenerative disorders throughout the body, especially in the brain, which uses more energy than any other organ [2]. Metabolic rate declines and ROS levels increase in neurological disorders, including stroke, Parkinson’s disease (PD), and Alzheimer’s disease (AD) [3]–[5]. Therefore, preserving brain function requires preserving mitochondrial integrity–that is, maintaining metabolic activity and ROS generation in a physiologically normal range.

Extensive research has explored neuroprotective strategies for preserving mitochondrial function and the treatment of neurological diseases. Methylene blue (MB) is one such treatment. Synthesized in 1886, MB has been used for more than a century to treat many diseases, such as malaria, methemoglobinemia, and cyanide poisoning [6], [7]. In the central nervous system, MB is a neuroprotectant against various insults *in vitro* [8]–[10]. MB, functioning as an alternative electron carrier, can accept electrons from NADH (mitochondrial complex I) and transfers them to cytochrome *c* (complex IV) via bypassing complex I/III blockage [11]–[13] (also see Figure 10 in Ref. [10]). Thus, MB can prevent electron leaking, increase mitochondrial oxidative phosphorylation, and reduce ROS overproduction under pathological conditions.

In line with this conclusion, a recent study found that MB attenuates behavioral, neurochemical, and neuropathological impairment in a PD model and significantly reduced cerebral ischemia reperfusion damage in a transient focal cerebral ischemia model [10]. MB slowed the progression of AD pathology and cognitive function decline in transgenic AD models and a clinical trial [14]–[16]. Hypometabolism in the posterior cingulate/retrosplenial cortex is a common feature in amnestic mild cognitive impairment and AD [17], [18]. In rats with metabolic lesions, MB treatment reduced metabolic lesion volume in the posterior cingulate/retrosplenial cortex, restored the function of the cingulo-thalamo-hippocampal network, and improved memory [19]. MB also enhances mitochondrial complex IV (cytochrome *c* oxidase) activity and therefore has been proposed as a metabolic enhancer that attenuates neurodegeneration induced by metabolic challenge [20], [21].

Despite the extensive studies of MB effects under neuropathological conditions, the action of MB on brain metabolic and vascular functions remains an object of study. Previous studies have used mainly nonneuronal cell lines, e.g., fibroblast [8] and mitochondria isolated from the heart [10] and liver [8]. In this study, we used a neuronal cell line (HT22 cells) to investigate MB effects on glucose uptake and mitochondria isolated from rat brains to investigate mitochondrial complex I–III activity. Further, we used noninvasive neuroimaging methods to identify MB effects on brain metabolism and hemodynamics *in vivo.* We used positron emission tomography (PET) to measure glucose uptake and magnetic resonance imaging (MRI) to measure cerebral blood flow (CBF). Cerebral metabolic rate of oxygen (CMRO_2_) was also determined. The purpose of the study was to use *in vitro* and *in vivo* studies to evaluate how MB affects cerebral glucose uptake, CMRO_2_, and CBF under both basal and inhibitory conditions in rats.

## Results

### In Vitro Assays

#### MB enhances complex I–III activity

**Figure 1:**
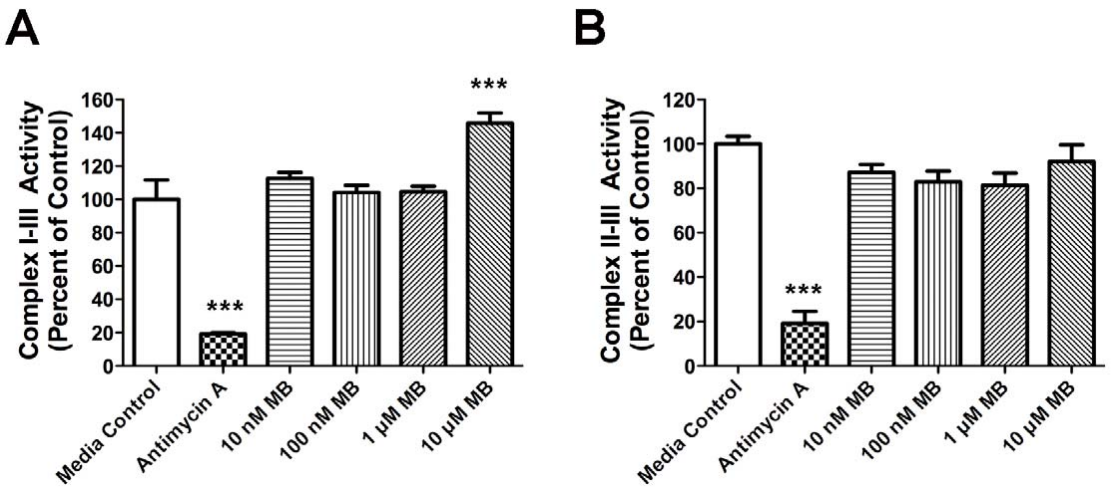
MB enhances mitochondrial complex I–III activity. MB enhances mitochondrial complex I–III activity (**A**), but not II–III (**B**) activity in mitochondria isolated from rat brains. Antimycin A, an inhibitor of complex III, significantly reduced complex I–III and II–III activities. Data are mean ± SD; ***, *p*<0.001.

To determine MB’s effect on the electron transport chain, we measured the rate of reduction of cytochrome *c*, using NADH as the electron donor. NADH oxidase (complex I) oxidizes NADH to NAD^+^ and transfers an electron to coenzyme Q10 (CoQ10). Cytochrome *c* reductase (complex III) oxidizes CoQ10 and reduces cytochrome *c*. With NADH as the electron donor, 10 µM MB increased the rate of reduction of cytochrome *c* (Figure 1A). However, when succinate was the electron donor, facilitating a transfer of electrons from succinate to CoQ10 by succinate dehydrogenase (complex II), followed by cytochrome *c* reductase, we saw no significant increase in the rate of cytochrome *c* reduction compared with control (Figure 1B). By contrast, antimycin A, an inhibitor for complex III, dramatically reduced complex I–III and II–III activities.

#### MB increases cellular oxygen consumption

**Figure 2:**
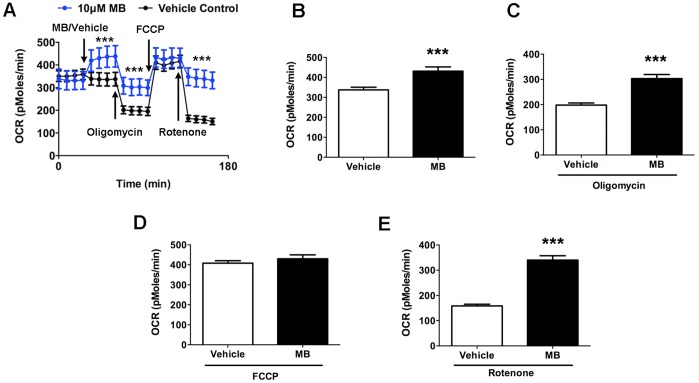
MB increases OCR in HT-22 cells. (**A**) We monitored cellular OCR with sequential administration of MB/vehicle, oligomycin, FCCP, and rotenone. Quantitative analysis of OCR shows that MB increases OCR under (**B**) normal, (**C**) ATP synthase inhibition (oligomycin), (**D**) FCCP, and (**E**) complex I inhibition (rotenone). Data are mean ± SD; ***, *p*<0.001.

We measured MB’s effect on the cellular oxygen consumption rate (OCR) of HT-22 cells by using an XF24 Flux Analyzer. We injected vehicle to serve as the control procedure. Figure 2A shows the timeline of the experiment, and Figures 2B–E show the quantitative measures. First, we measured OCR for 30 min to establish a baseline reading, and then we injected 10 µM MB or vehicle into half the wells. OCR in MB-treated cells significantly increased compared with that in vehicle-treated cells (Figure 2B). With the addition of oligomycin (ATP synthase inhibitor), OCR decreased in both vehicle- and MB-treated cells. MB-treated cells remained at a significantly higher OCR than did those injected with vehicle (Figure 2C). With the addition of the membrane uncoupler carbonyl cyanide *p*-trifluoromethoxyphenyl hydrazone (FCCP), we obtained maximum OCR, with no difference between vehicle- and MB-treated groups (Figure 2D). Finally, adding rotenone (complex I inhibitor) caused a decrease in OCR; however, MB-treated cells had significantly greater OCR than did those treated with vehicle (Figure 2E).

#### MB increases cellular glucose uptake

**Figure 3:**
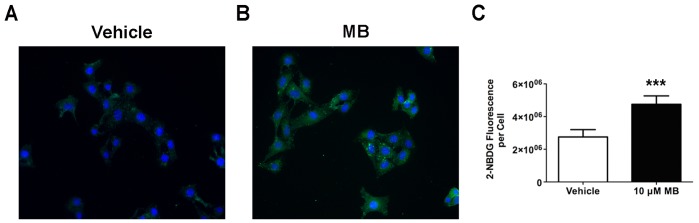
MB increases glucose uptake in HT-22 cells. Representative live cell images show glucose uptake in (**A**) vehicle- and (**B**) MB-treated HT-22 cells. (**C**) Quantitative analysis indicates a significant increase of glucose uptake upon MB treatment at 10 µM. Data are mean ± SD; ***, *p*<0.001.

We used a fluorescent d-glucose analog, 2-[*N*-(7-nitrobenz-2-oxa-1,3-diazol-4-yl) amino]-2-deoxy-d-glucose (2-NBDG), to measure glucose uptake in HT-22 cells. Uptake of 2-NBDG occurs through the same mechanisms as glucose, and intracellular breakdown of 2-NBDG occurs over an extended time, allowing quantitative analysis of cellular 2-NBDG uptake [22]. After 30 min of glucose deprivation, we incubated cells in 10 µM MB and 100 µM 2-NBDG in glucose-free media for 5 min and then measured the intracellular 2-NBDG. Intracellular 2-NBDG increased significantly in the presence of MB (Figure 3A and B). We have normalized the quantification to account for total cell numbers in mean 2-NBDG fluorescence per cell (Figure 3C).

### *In Vivo* Measurements

#### MB enhances global glucose uptake and CMRO_2_

**Figure 4:**
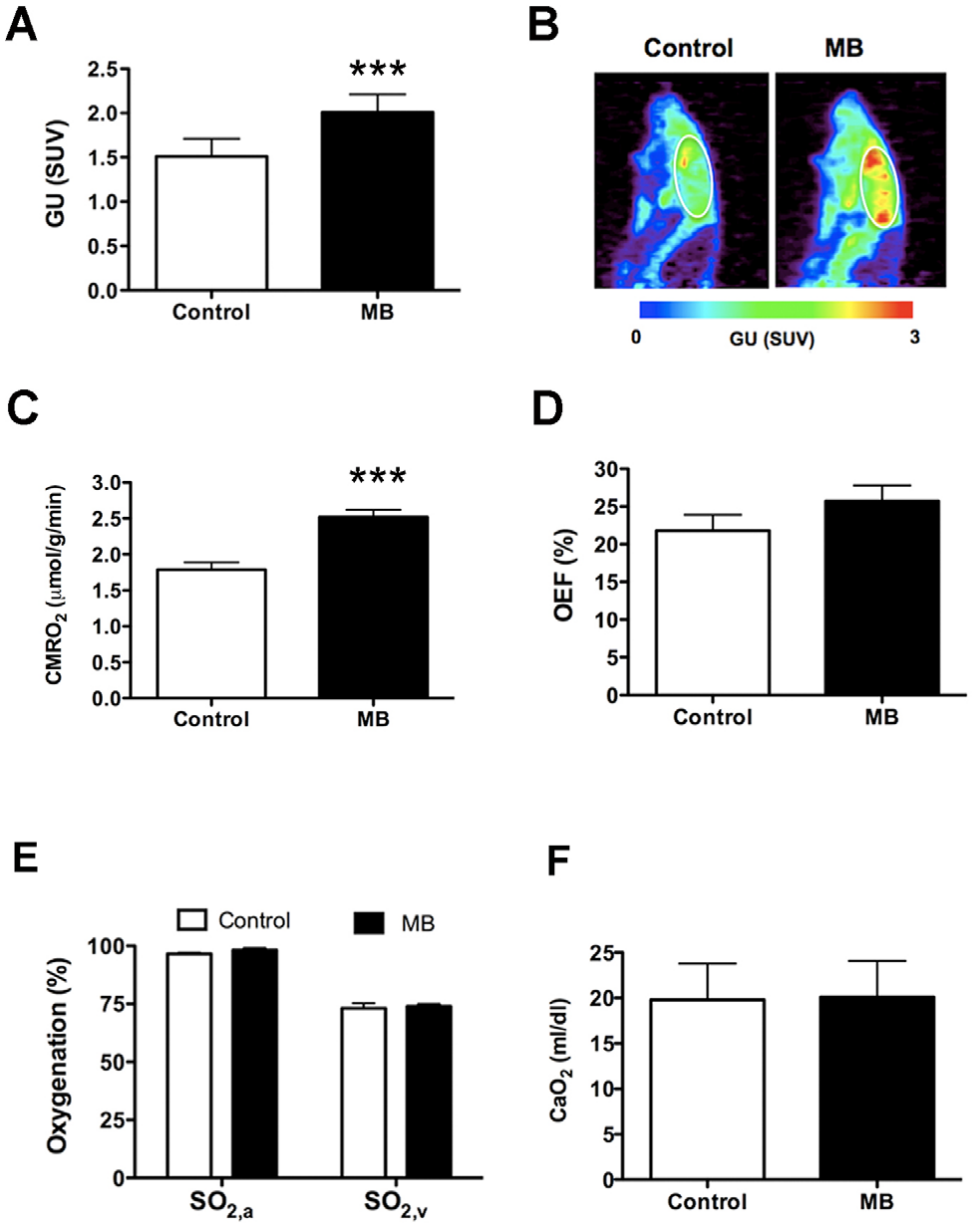
MB enhances glucose uptake and CMRO_2_ under normoxic conditions. (**A**) Averaged values of glucose uptake (GU). (**B**) Glucose uptake maps from a single rat under control (normoxia) and MB (normoxia + MB) conditions. (**C**) Averaged values of global CMRO_2_. (**D**) Averaged values of OEF. (**E**) Arterial (SO_2,a_) and venous (SO_2,v_) oxygenation. (**F**) Oxygen content (CaO_2_). Data are mean ± SD; ***, *p*<0.001.

Figure 4A shows averaged values of global glucose uptake under air-breathing conditions; Figure 4B shows representative glucose uptake maps. We detected significant increase of glucose uptake with MB (27% ±8% increase) compared with that in the controls. Similarly, global CMRO_2_ was 39% ±11% higher in rats with MB treatment (Figure 4C), despite the insignificant change of oxygen extraction fraction (OEF) (Figure 4D). However, MB administration did not significantly affect arterial (SO_2,a_) and venous (SO_2,v_) oxygenation (Figure 4E) or the oxygen content (CaO_2_) (Figure 4F).

#### MB enhances global and regional CBF that involves neurological pathology

**Figure 5:**
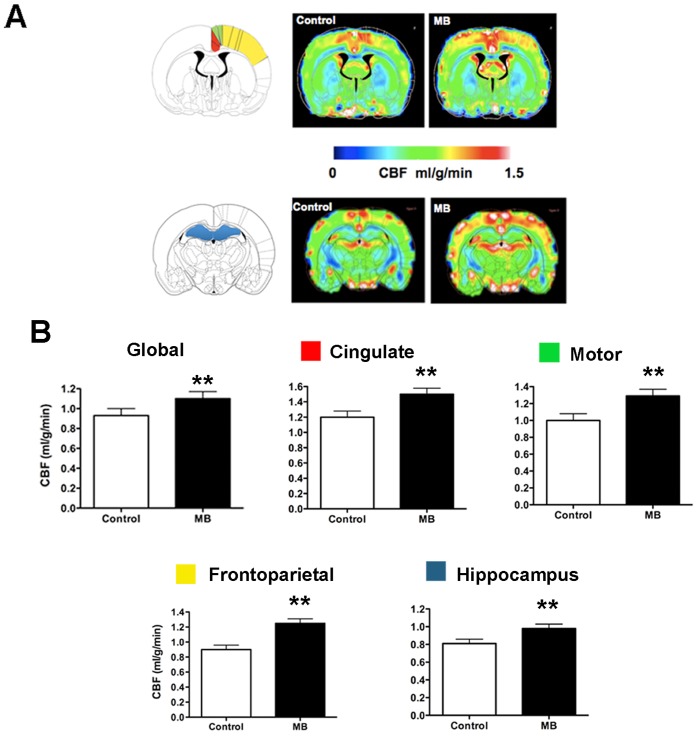
MB enhances global and regional CBF under normoxic **conditions.** (**A**) Brain regions related to PD and AD pathology and the CBF maps under control (normoxia) and MB (normoxia + MB) conditions. Red, cingulate cortex; green, motor cortex; yellow, frontoparietal cortex; blue, hippocampus. (**B**) Global CBF and regional CBF in cingulate, motor, frontoparietal cortices and hippocampus. Data are mean ± SD; **, *p*<0.01.

Global CBF increased 18% ±6% with MB administration (Figure 5A and B). The higher spatial resolution of using MRI to measure CBF enabled us to quantify CBF regionally. Our regional CBF analysis included regions involved in the pathology for AD and PD, including the frontoparietal cortex, cingulate cortex, motor cortex, and hippocampus. Figure 5A shows the locations of the cingulate, motor, and frontoparietal cortices; the hippocampus; and the corresponding CBF maps obtained under control (normoxia; air only) and MB treatment conditions. With MB, regional CBF in all the areas was significantly higher than under basal conditions (Figure 5B).

#### MB preserves global glucose uptake, CBF, and CMRO_2_ under hypoxia

**Figure 6:**
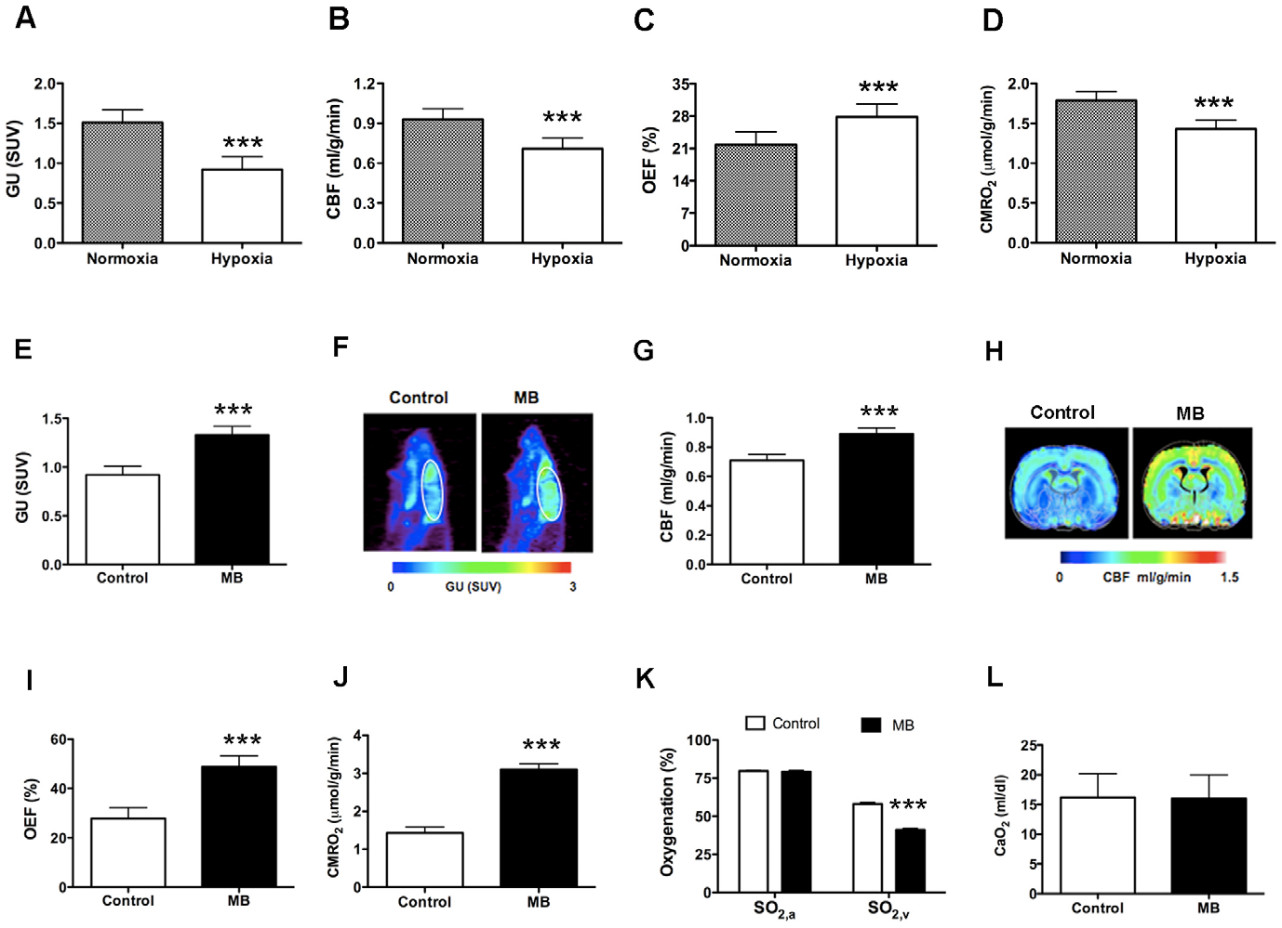
Glucose uptake, CBF, and CMRO_2_ were reduced under hypoxic conditions but enhanced by MB treatment. Comparison between normoxia and hypoxia of (**A**) glucose uptake, (**B**) CBF, (**C**) OEF, and (**D**) CMRO_2_. Comparison between control (hypoxia) and MB treatment (hypoxia + MB) of (**E, F**) glucose uptake (values and maps). (**G, H**) CBF (values and maps), (**I**) OEF, (**J**) CMRO_2_, (**K**) arterial (SO_2,a_) and venous (SO_2,v_) oxygenation. (**L**) Oxygen content (CaO_2_). Data are mean ± SD; ***, *p*<0.001.

Under hypoxic conditions, relative to normoxia, global glucose uptake decreased by 39% ±12% (Figure 6A), CBF decreased by 24% ±9% (Figure 6B), OEF increased by 28% ±6% (Figure 6C), and CMRO_2_ decreased by 22% ±4% (Figure 6D). After MB injection, glucose uptake increased by 55% ±18% (Figure 6E and F), CBF increased by 25% ±10% (Figure 6G and H), OEF increased by 76% ±19% (Figure 6I), and CMRO_2_ increased by 121% ±29% (Figure 6J). Glucose uptake, CBF, and CMRO_2_ values obtained under hypoxia in the presence of MB were similar to those obtained under the baseline (normoxia) condition. MB preserved glucose uptake, CBF, and CMRO_2_ in normal physiological ranges in hypoxic conditions. However, MB did not significantly affect SO_2,a_ but decreased SO_2,v_ (Figure 6K). CaO_2_ remained the same with MB injection (Figure 6L).

#### MB has no significant effect on PO_2_, PCO_2_, and hematocrit

**Figure 7:**
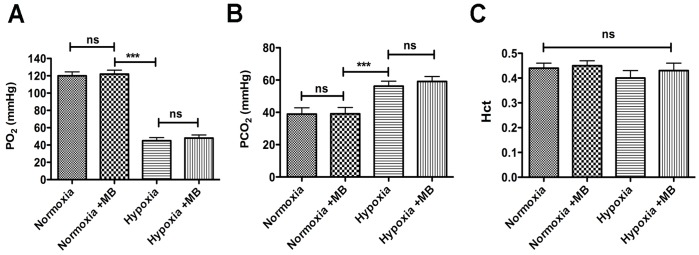
MB did not affect PO_2_, PCO_2_, and hematocrit (Hct). (**A**) PO_2_ decreased under hypoxia but did not change with MB treatment (under either condition). (**B**) PCO_2_ increased under hypoxia but did not change with MB treatment (under either condition). (**C**) Hematocrit was not affected by gas type or MB treatment. Data are mean ± SD; ***, *p*<0.001; ns, not significant.

Oxygen partial pressure in the blood (PO_2_) was significantly reduced and carbon dioxide partial pressure (PCO_2_) was significantly increased under hypoxia compared with normoxia. However, MB injection did not affect PO_2_ and PCO_2_ under either normoxic or hypoxic conditions (Figure 7A and B). However, hematocrit values were not significantly different among the four conditions (Figure 7C), suggesting that MB can effectively alter oxygen and glucose metabolism without disturbing the O_2_ and CO_2_ tension as well as the packed cell volume (PCV; i.e., red blood cell volume).

## Discussion

Using multimetric neuroimaging systems, we measured quantitative glucose uptake, CMRO_2_, and CBF, and we evaluated MB effects on *in vivo* brain metabolism and hemodynamics. MB enhanced global glucose uptake, CMRO_2_, and CBF under both basal (normoxic) and inhibitory (hypoxic) conditions. MB also significantly increased regional CBF in brain areas involved in AD and PD pathology, including the hippocampus and the cingulate, motor, and frontoparietal cortices. MB treatment did not affect PO_2_, PCO_2_, and hematocrit levels. Our *in vivo* results are in good agreement with the *in vitro* assays, which show dramatic elevations of cellular oxygen consumption and glucose uptake in the HT-22 hippocampal cell line under normal conditions. In the mitochondria isolated from rat brain, MB accepts electrons from NADH and significantly increases mitochondrial complex I–III activity in both basal and inhibitory conditions. Our *in vitro* findings are consistent with those in the literature [11]–[13].

Under normoxia, acute MB treatment caused parallel increases in CMRO_2_, glucose uptake, and CBF. We expected the MB-induced increases in CMRO_2_ owing to the increased activity in the mitochondrial electron transport chain. The CMRO_2_ increases resulted mainly from elevated oxygenation (i.e., increased CBF) rather than from oxygen extraction (i.e., OEF), because CBF dramatically increased, whereas OEF remained unchanged. The finding of MB-evoked increases in glucose uptake was in good agreement with literature findings that MB can stimulate glucose metabolism, possibly via a single glucose transporter, GLUT1 [23]. Taken together, the increases in CMRO_2_ and glucose uptake suggest that MB can elevate oxygen consumption for the oxidative phosphorylation of glucose to increase ATP production [10]. We speculated that the increased demand for delivery of glucose and oxygen through circulation may upregulate the increases in CBF. Future studies need to examine the mechanism of MB-induced CBF changes. Nonetheless, the MB-induced parallel increases in CMRO_2_, glucose uptake, and CBF are consistent with literature findings showing that basal neurovascular and neurometabolism are tightly coupled [24], [25].

We also observed flow–metabolism coupling during hypoxia: CBF, glucose uptake, and CMRO_2_ decreased under hypoxic conditions. Our results were consistent with those of previous observations that hypoxia did not evoke CBF increases [26]. With reduced CBF, OEF increased, as expected, to maintain oxygen availability for brain tissue [27]. The reductions of CMRO_2_ and glucose uptake suggest reduced overall energy metabolism of the animal under hypoxia. Nonetheless, MB could attenuate the metabolic and hemodynamic deficits due to hypoxia. Therefore, under hypoxic conditions, MB elevated and preserved glucose uptake, CBF, OEF, and CMRO_2_.

Our results suggest that MB protects brain tissue in low-oxygen environments *in vivo*, which could be an important factor of neuroprotection under stroke. In ischemic stroke, however, brain tissue has low or no CBF in addition to low oxygen. Here we showed that MB can preserve CMRO_2_ primarily by extracting oxygen from the blood by increasing OEF (49% increase) compared with elevated CBF (25% increase)., While reperfusion treatment (i.e., increasing CBF) has been used to salvage brain tissue under ischemic stroke, the procedure could also cause inflammation and oxidative damage by inducing oxidative stress. In a previous study, we showed *in vitro* that MB can significantly reduce cerebral ischemic reperfusion damage by rerouting electrons, thereby reducing ROS and oxidative stress. Therefore, MB-treated brain tissue has significantly less ischemic lesion than the control samples [10]. In a future study, we will use multimetric neuroimaging systems to characterize MB’s effects on ischemic stroke and reperfusion damage *in vivo*.

Our data are also in line with the literature that MB protects against effects of AD and PD [10], [14]. For example, we observed MB-enhanced complex I–III activity. Mitochondrial complex I deficit is a common feature in PD and AD [1], [28], [29]. The enhanced complex I–III activity by rerouting electrons from complex I to III can thus alleviate the disease burden. We also observed MB-enhanced CBF in the brain regions involved in AD and PD pathology. The MB-induced increases in CBF (and therefore in metabolism) suggest that MB can maintain normal hemodynamics and metabolism in brain regions associated with cognitive functions. These findings are consistent with those of previous reports: MB-treated rats with AD had significantly improved memory and learning functions [15], [16], and MB-treated rats with PD performed better on rotarod tests [10].

Collectively, administering MB *in vivo* appears to benefit the cerebral metabolic and vascular functions. However, MB shows a hermetic dose-dependent response, with opposite effects at low and high doses ([30] and review in [21]). At low doses (e.g., 0.5–4 mg/kg of body weight), MB is an alternative electron carrier in the mitochondrial electron transport chain, with unparalleled antioxidant and cell respiration–enhancing properties that affect nervous system function. By contrast, high doses of MB (e.g., >10 mg/kg) show adverse effects. Rodents treated with 4 mg of MB per kilogram showed dramatically enhanced memory, whereas larger doses (e.g., 50 and 100 mg/kg) caused adverse effects [30]. Using a low dose of MB in the present study (0.5 mg/kg), we observed beneficial effects of MB on *in vivo* brain metabolism and hemodynamics.

For future studies, we will characterize MB treatment efficacy *in vivo* by using neuroimaging methods in disease models, including ischemic stroke, PD, and AD. We have established these models and studied the MB effects *in vitro* [10]. The combination of *in vitro* assays and *in vitro* metabolic–hemodynamic profile will give us a comprehensive understanding of how these neurological diseases respond to treatment.

Our study showed that acute MB treatment could enhance brain metabolism and hemodynamics under normoxic and hypoxic conditions. Using noninvasive, multimetric neuroimaging methods, we investigated the MB’s effect on *in vivo* brain metabolism and hemodynamics. We corroborated our *in vivo* results by comparing with those obtained under similar conditions from *in vitro* assays. These neuroimaging methods can offer longitudinal and noninvasive investigation of treatment efficacies of MB in neurological disorders *in vivo*. MB is a U.S. Food and Drug Administration–approved drug for other indications and has well-established safety profiles; therefore, clinical trials could use neuroimaging to readily explore MB’s efficacy with other neurological disorders. The imaging-based evaluation of MB treatment efficacy will have profound implications for future studies of neurodegenerative disorders.

## Materials and Methods

### *In Vitro* Assays

#### Isolation of brain mitochondria

We isolated mitochondria from 3-month-old Sprague-Dawley rat brains, as previously described by Sims and colleagues [31]. In brief, we used a razor blade to mince the brains and placed them in 15 mL of isolation buffer (0.32 M sucrose, 1 mM K2EDTA, 10 mM Tris base [pH 7.1]). We placed the isolation buffer containing brain fragments in a glass homogenizer and broke them up with 10 strokes. We centrifuged the homogenized solution for 5 min at 1,330× *g*. We collected and recentrifuged the supernatant at 21,200× *g* for 10 min. We collected and resuspended the pellet in 10 mL of isolation buffer containing 0.02% digitonin. We broke up the pellet and mixed the solution for 10 min at 4°C. Afterward, we centrifuged the suspension at 6,900× *g* for 10 min. We washed off the fluffy white layer surrounding the mitochondria pellet (brown) and resuspended it in 12% Percoll solution (0.32 M sucrose, 1 mM K2EDTA, 10 mM Tris base [pH 7.1], 12% Percoll). We centrifuged the Percoll solution containing the mitochondria pellet at 6,900× *g* for 10 min. We removed the supernatant and washed the pellet once in isolation buffer. We then centrifuged the solution for 10 min at 6,900× *g* and stored the pellet at –80°C until use [32]. Before use, we sonicated the mitochondria three times for 30 s to fractionate mitochondrial membranes.

#### Mitochondrial complex activity assay

For the complex I–III assay, we added mitochondria membrane fractions to 50 mM phosphate buffer (pH 7.4) containing 2 mM MgCl_2_, 2 mM KCN, 80 µM oxidized cytochrome *c*, and 4 µM NADH. We monitored changes in absorbance at 550 nm with a Tecan Infinite F200 plate reader. We added 2 µg of antimycin per milliliter to inhibit complex III activity. For the complex II–III assay, we added mitochondria membrane fractions to 50 mM phosphate buffer (pH 7.4) containing 20 mM succinate, 500 µM EDTA, 2 mM KCN, 30 µM oxidized cytochrome *c*, and 2 µg of rotenone per milliliter. We monitored changes in absorbance at 550 nm with a Tecan Infinite F200 plate reader. We added 2 µg of antimycin per milliliter to inhibit complex III activity. We compared the control and MB groups with different dosage by one-way analysis of variance with tukey post hoc test.

#### Cell culture

HT-22 cells, a murine hippocampal cell line that is a subclone of HT4 cells. We maintained cells in Dulbecco’s modified Eagle medium (HyClone, Logan, UT) supplemented with 10% fetal bovine serum (HyClone) and penicillin–streptomycin solution in monolayers in 10-cm Greiner tissue culture dishes (Orlando, FL) under standard cell culture conditions (5% CO_2_, 95% air). We changed the medium three times weekly and back-cultured at confluence (every 3–5 days). We observed cells with a phase-contrast microscope (Zeiss Observer Z1). We used HT-22 cells at passages 10–30.

#### Glucose uptake

We determined the effect of MB on glucose uptake by using the 2-NBDG assay. We plated HT-22 cells onto 25-mm coverslips at a density of 2.5×10^4^ cells/coverslip and allowed them to attach overnight. The next day, we incubated cells in glucose-free Krebs Ringer HEPES (KRH) buffer (129 mM NaCl, 5 mM NaHCO_3_, 4.8 mM KCl, 1.2 mM KH_2_PO_4_, 1 mM CaCl_2_, 1.2 mM MgCl_2_, 10 mM HEPES) for 30 min. Afterward, we incubated cells for 5 min at 37°C in KRH buffer containing 100 µM 2-NBDG and specific concentrations of MB. We washed the cells three times in KRH buffer and mounted them on a coverslip. We took images with a Zeiss Observer Z1 microscope. We used ImageJ software to quantify the 2-NBDG by dividing the cellular fluorescence by the total cell number. The number of cells per group was 319 for control and 359 for 10 µM MB. We compared the vehicle- and MB-treated groups with the paired *t* test.

#### Cellular bioenergetics (Seahorse)

We plated HT-22 cells at a density of 30,000/well on an XF24 plate. Cells attached overnight, and we exchanged the media 1 h before the assay for XF24 media. We diluted rotenone (final concentration, 0.1 µM), FCCP (final concentration, 0.3 µM), and oligomycin (final concentration, 1 µg/ml) into XF24 media and loaded into the accompanying cartridge. Injection of drugs into the medium occurred at the time points specified. We monitored oxygen consumption with a Seahorse Bioscience XF24 Extracellular Flux Analyzer. We compared the vehicle- and MB-treated groups with the paired *t* test.

### *In Vivo* Measurements

#### Animal preparation

We used male Sprague-Dawley rats (250–300 g) for the study. We anesthetized rats with 4.0% isoflurane for induction and used a face mask to maintain the level at a 1.2% isoflurane–air mixture. We continuously monitored respiration rate (90–130 bpm) and rectal temperature (37°C ±0.5°C). We recorded heart rate and blood oxygen saturation level (SaO_2_) with a MouseOx system (STARR Life Science, Oakmont, PA) and maintained these parameters within reference physiological ranges. To administer MB, we inserted an intravenous line into the tail vain. We performed all animal experiments with the approval of the Institutional Animal Care and Use Committee of the University of Texas Health Science Center at San Antonio.

#### Glucose uptake measurement

We injected 0.5 mCi of fluorodeoxyglucose (^18^F-FDG) dissolved in 1 mL of physiologic saline through the tail vein. We injected MB 10 min before administering ^18^F-FDG. We allowed enough time (approximately 40 min) for ^18^F-FDG uptake before scanning. We then moved the rat to the scanner bed (Focus 220 MicroPET; Siemens, Nashville, TN) and placed the rat in the prone position. We acquired emission data for 20 min in a three-dimensional list mode with intrinsic resolution of 1.5 mm. For image reconstruction, we used a Fourier algorithm to rebin the 3-D PET data into multiple frames of 1-s duration. After rebinning the data, we reconstructed a 3-D image for each frame by using a 2-D filtered back-projection algorithm. We applied decay and dead time corrections to the reconstruction process. We determined glucose uptake by using the mean standardized uptake value equation: SUV = (*A* × *W*)/*A*_inj_, where *A* is the activity of the region of interest (i.e., brain region), *W* is the body weight of the mice, and *A*_inj_ is the injection dose of ^18^F-FDG [33].

#### CBF measurement

We measured quantitative CBF (in milliliters per gram per minute) by using the MRI-based continuous arterial spin labeling (CASL) techniques [34], [35] on a horizontal 7-T/30-cm magnet and a 40-G/cm BGA12S gradient insert (Bruker, Billerica, MA). We placed a small circular surface coil (inside diameter, 1.1 cm) on top of the head and placed a circular labeling coil (inside diameter, 0.8 cm), built into the cradle, at the heart position for CASL. We positioned the two coils parallel to each other, separated by 2 cm center to center, and actively decoupled them. We acquired paired images in an interleaved fashion with the following parameters: field of view = 25.6×25.6 mm^2^, matrix = 128×128, slice thickness = 1 mm, nine slices, labeling duration = 2100 ms, TR = 3,000 ms, and TE = 20 ms. CASL image analysis employed codes written in Matlab [34], [35] and STIMULATE software (University of Minnesota) to obtain CBF.

#### Oxygen consumption measurement

We inserted polyethylene 50 tubings into the femoral artery and jugular vein. We took blood samples to measure oxygenation from the arterial (SaO_2,a_) and venous (SaO_2,v_) blood with a blood gas analyzer (Radiometer ABL5, Copenhagen). We determined OEF with the equation (SaO_2,a_ – SaO_2,v_)/SaO_2,a_. We then calculated global CMRO_2_ with the equation OEF × CBF × CaO_2_ (CaO_2_ is the oxygen content). We also obtained PO_2_, PCO_2_, and CaO_2_ values through blood gas measurements.

#### Hematocrit measurement

We also determined the MB effect on hematocrit. After the blood gas measurement, we took residual venous blood with a heparinized microhematocrit capillary tube (Fisher Scientific, Pittsburgh, PA) and centrifuged it for 3 min. We then determined hematocrit by the ratio between the PCV (i.e., red blood cell volume) and the whole blood (plasma + PCV).

#### Under normoxic baseline condition (*N* = 6)

We measured glucose uptake, CBF, and CMRO_2_ with three different sets of rats on three different days (e.g., glucose uptake on day 1 with group 1; CBF on day 2 with group 2; CMRO_2_ on day 3 with group 3). Baseline (normoxia; air only) measurements were followed by MB administration (normoxia + MB). We injected MB at a dose of 0.5 mg/kg through the tail vein at a rate of 0.1 mg/kg/min. We waited 10 min for MB uptake before the measurements [36]. Because of the long half-life of the ^18^F-FDG PET tracer (110 min), we measured glucose uptake on each condition on different animals (e.g., baseline measurement on rat 1 and MB measurement on rat 2; therefore, the PET study used a total of 12 rats).

#### Under hypoxic condition (*N* = 6)

We measured glucose uptake, CBF, and CMRO_2_ under hypoxia on the same set of animals as for normal condition 1 week later to ensure MB clearance. We performed these three experiments on three different days as well. We gave 10% O_2_ (and balanced N_2_) during the hypoxic condition. We took measurements after oxygen saturation (SaO_2_) reduced to 65–70%. Similar to the normal condition, baseline (10% O_2_) measurements were followed by MB administration (MB +10% O_2_). We allowed 10 min between the two measurements. We also measured glucose uptake (with and without MB) on different rats.

We performed the *in vivo* study in a blinded fashion, with one group of investigators executing experiments (F.D. and S.H) and a separate group analyzing data (A.L. and A.B.). Those analyzing the data were unaware of which session that animal had received MB until after the experiment.

#### Statistical analysis

We compared all the measured variables of the *in vivo* data (two gas types and MB treatment) with two-way analysis of variance. We used the Newman–Keuls test for post hoc analysis.
